# Supplementary material for: Testosterone Serum Levels Are Related to Sperm DNA Fragmentation Index Reduction after FSH Administration in Males with Idiopathic Infertility
Source: Biomedicines. 2022 Oct 17;10(10):2599. doi: 10.3390/biomedicines10102599 (PMC9599665; doi:10.3390/biomedicines10102599)

Supplementary table 1. Correlation analyses among available variables at baseline.

		BMI	sDF index	Testosterone	LH	FSH	SHBG	Inhibin B	AMH	Semen volume	Sperm concentration	Total sperm number	Progressive sperm motility	Normal morphology
**Age**	Rho	-0.032	-0.177	-0.061	0.048	-0.002	-0.060	-0.031	0.012	0.024	0.091	0.087	0.099	-0.093
	p-value	0.716	0.005	0.459	0.595	0.985	0.368	0.648	0.858	0.788	0.309	0.328	0.266	0.296
**BMI**	Rho		0.008	-0.206	0.085	0.094	0.094	-0.409	-0.146	0.078	-0.032	-0.005	-0.016	0.184
	p-value		0.925	0.019	0.348	0.299	0.299	0.005	0.117	0.381	0.718	0.952	0.858	0.039
**sDF index**	Rho			-0.006	-0.134	0.021	0.009	0.000	0.008	0.161	-0.325	-0.199	-0.035	0.294
	p-value			0.940	0.137	0.818	0.896	0.997	0.905	0.069	**<0.001**	0.025	0.698	**0.001**
**Testosterone**	Rho				0.362	0.082	0.061	0.247	0.069	-0.059	-0.059	-0.036	0.047	0.102
	p-value				**<0.001**	0.362	0.362	**<0.001**	0.305	0.508	0.512	0.685	0.600	0.253
**LH**	Rho					0.395	0.118	0.036	-0.088	-0.070	-0.163	-0.138	-0.044	0.051
	p-value					**<0.001**	0.076	0.590	0.193	0.441	0.070	0.127	0.626	0.572
**FSH**	Rho						-0.068	0.281	-0.039	-0.024	-0.241	-0.207	0.026	0.145
	p-value						0.642	**<0.001**	0.562	0.788	0.007	0.021	0.772	0.107
**SHBG**	Rho							0.033	0.177	-0.067	0.022	0.000	-0.116	-0.160
	p-value							0.721	0.008	0.318	0.740	0.998	0.083	0.016
**Inhibin B**	Rho								0.102	-0.067	0.046	0.007	-0.086	-0.047

	p-value								0.210	0.320	0.493	0.916	0.205	0.483
**AMH**	Rho									0.037	0.003	0.026	-0.003	-0.136
	p-value									0.581	0.962	0.703	0.968	0.042
**Semen Volume**	Rho										0.27	0.597	0.070	0.047
	p-value										**0.002**	**<0.001**	0.434	0.598
**Sperm concentration**	Rho											0.917	0.279	-0.359
	p-value											**<0.001**	**0.001**	**<0.001**
**Total sperm number**	Rho												0.278	-0.253
	p-value												**0.001**	0.004
**Progressive sperm motility**	Rho													-0.061
	p-value													0.491

*[Footnotes to **supplementary table 1**: AMH: anti-Mullerian hormone; BMI: body mass index; FSH: follicle stimulating hormone; LH: luteinizing hormone; sDF: sperm DNA fragmentation; SHBG: sex hormone binding globulin]*

Supplementary table 2. Correlation analyses among available variables after follicle stimulating hormone (FSH) administration.

		BMI	sDF	Testosterone	LH	FSH	SHBG	Inhibin B	AMH	Semen volume	Sperm concentration	Total sperm number	Progressive sperm motility	Normal morphology
**Age**	Rho	0.069	0.090	0.082	0.095	-0.215	0.025	0.054	-0.033	-0.061	-0.204	-0.186	-0.054	0.032
	p-value	0.472	0.277	0.321	0.323	0.024	0.719	0.500	0.646	0.524	0.030	0.047	0.567	0.735

**BMI**	Rho		-0.011	-0.153	-0.076	-0.047	-0.208	-0.132	-0.187	0.119	0.011	0.018	0.065	0.058
	p-value		0.911	0.108	0.436	0.631	0.033	0.197	0.071	0.244	0.913	0.861	0.525	0.572
**sDF**	Rho			-0.327	0.070	0.021	-0.144	0.004	0.006	-0.138	-0.316	-0.267	-0.189	-0.163
	p-value			**0.002**	0.464	0.829	0.035	0.961	0.936	0.145	**0.001**	**0.003**	0.045	0.085
**Testosterone**	Rho				0.272	-0.081	0.326	0.013	0.097	-0.045	-0.164	-0.148	-0.121	0.179
	p-value				**0.004**	0.395	**<0.001**	0.867	0.172	0.637	0.082	0.116	0.202	0.057
**LH**	Rho					0.351	0.017	-0.152	0.097	-0.073	-0.213	-0.190	-0.152	0.070
	p-value					**<0.001**	0.808	0.056	0.169	0.467	0.033	0.057	0.130	0.488
**FSH**	Rho						-0.052	0.307	0.028	0.085	-0.112	-0.057	-0.096	0.025
	p-value						0.447	**<0.001**	0.698	0.399	0.266	0.570	0.339	0.807
**SHBG**	Rho							0.079	0.084	0.098	0.011	0.006	-0.073	-0.092
	p-value							0.319	0.234	0.163	0.873	0.931	0.299	0.191
**Inhibin B**	Rho								-0.214	0.165	0.057	0.118	0.055	0.149
	p-value								0.007	0.043	0.491	0.148	0.505	0.068
**AMH**	Rho									0.013	-0.068	-0.068	0.026	-0.069
	p-value									0.859	0.346	0.345	0.723	0.338
**Semen Volume**	Rho										0.341	0.605	0.204	0.050

	p-value										**<0.001**	**<0.001**	0.031	0.601
**Sperm concentration**	Rho											0.945	0.568	-0.264
	p-value											**<0.001**	**<0.001**	**0.005**
**Total sperm number**	Rho												0.537	-0.209
	p-value												**<0.001**	0.026
**Progressive sperm motility**	Rho													-0.189
	p-value													0.044

*[Footnotes to **supplementary table 2**: AMH: anti-Mullerian hormone; BMI: body mass index; FSH: follicle stimulating hormone; LH: luteinizing hormone; sDF: sperm DNA fragmentation; SHBG: sex hormone binding globulin]*

Supplementary figure 1. PRISMA diagram

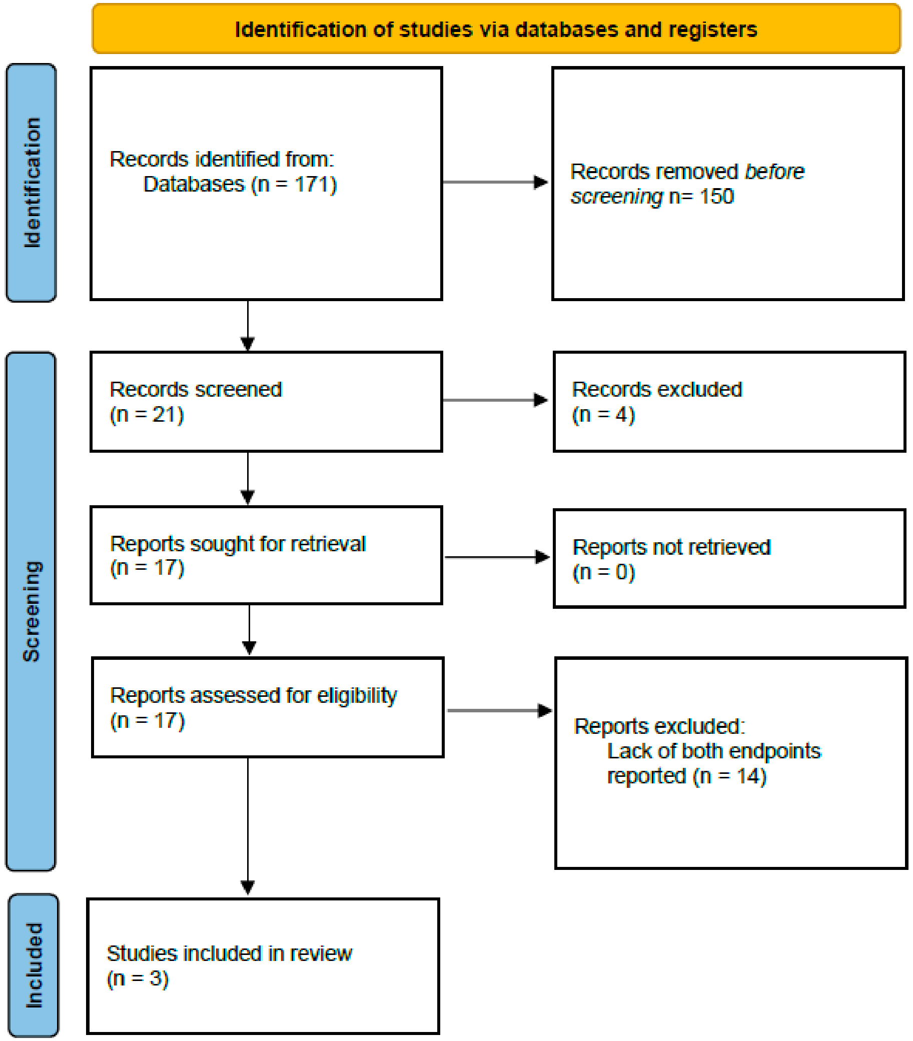

Supplement: Supplementary file 1 [file biomedicines-10-02599-s001.zip › biomedicines-1872409-supplementary.pdf]
